# Editorial: Environmental and socio-economic drivers of migrant farmworkers' health

**DOI:** 10.3389/fpubh.2024.1440739

**Published:** 2024-08-13

**Authors:** Federico Castillo, David Lopez-Carr, Armando Sanchez

**Affiliations:** ^1^Department of Environmental Science, Policy and Management, University of California, Berkeley, Berkeley, CA, United States; ^2^Department of Geography, College of Letters and Science, University of California, Santa Barbara, Santa Barbara, CA, United States; ^3^Instituto de Investigaciones Económicas, Universidad Nacional Autónoma de México, Mexico City, Mexico

**Keywords:** farmworkers, extremes, health, agriculture, environmental justice

Migrant farm workers are among the most vulnerable segments of the labor force, not only in the United States, but around the world (1–3). Migrant farm worker social vulnerability is translated into poor health among other deleterious outcomes. For example, during the COVID-19 pandemic farmworkers (including migrant farmworkers) were deemed “essential” to society yet, they were not provided with adequate resources to cope with COVID-19 health and socio-economic impacts.

The purpose of this Research Topic titled “*Environmental and socio-economic drivers of migrant farmworker health*” is to present a comprehensive analysis and latest research findings on topics related to migrant farm workers health and wellbeing. By recognizing that migrant farmworkers are an important component in the food supply chain, the Research Topic touches not only on migrant farm worker health outcomes but the on-farm and off-farm determinants of health. Migrant farm workers are impacted by a myriad of environmental threats that, coupled with institutional arrangements that impact them negatively, result in serious social costs to society such as reduction in their labor productivity as well as in farm productivity (4).

The Research Topic recognizes that to analyze how environmental drivers impact migrant farm workers we must approach the analysis from a multidisciplinary perspective. The Research Topic contributors include scholars in the public health, economics, geography, nutrition, public policy, and medical fields, among others. The authors used a variety of data collection methods including in person interviews and use of secondary data. Research methods using qualitative and quantitative data analysis complement the theoretical and empirical underpinnings of the results presented in the Research Topic. The articles present to the reader a wide variety of policy suggestions that address how to improve the social welfare of migrant farm workers. Taken as whole, this Research Topic allows the reader, whether the reader is part of the academic sector, policy or non-profit sector, to understand how public health and other disciplines research can effectively contribute to improve the wellbeing of migrant farmworkers.

The literature supports the notion that the COVID-19 pandemic impacted disproportionally the agricultural labor sector not only in California, but also in other regions of the US and other countries (5, 6). In this Research Topic, Mora et al. (5) analyze the impact of COVID-19 on farmworkers in Michoacan, Mexico. As with other articles in this Research Topic, Mora et al. (5) complements the analysis of rates of COVID-19 infection among avocado farmworkers with impacts on mental health and food security, emphasizing the need to understand the impact of COVID-19 on farmworkers as part of a complex socio-economic-health dynamic that needs to be understood in a holistic manner.

Using data generated by the Mexican Immigration to California: Agricultural Safety and Acculturation (MICASA), the Pasos Saludables and the Pasos studies, Matias et al. present an analysis of the prevalence of diet-related chronic health outcomes in 1,300 migrant and non-migrant farm workers from California. Health outcomes analyzed included obesity, elevated waist circumference, high blood pressure and high total cholesterol. Their results show that women are at greater risk of obesity and higher waist circumference. Single, divorced, or widowed farm workers had reduced odds of obesity and elevated waist circumference. These results, together with other findings, demonstrate the need to target interventions along age, gender, and other socio-economic characteristics if those interventions are to be effective.

Using data generated by the Agricultural Worker Survey (NAWS) and collected in six US regions, Soto et al. present an innovative analysis for professionals of health and related fields to improve migrant farm workers' access to health care by considering barriers to health care access ingrained in the agricultural industry. Understanding how these barriers form and persist can help to improve health outcomes for migrant farm workers. After adjusting for age, gender, income, and other socio-economic variables their logistic regression model shows that health outcomes vary across regions. Their results show that while asthma rates are lower in the Southwest region relative to the other regions under consideration, the rate of diabetes shows opposite results. As with the study by Matias, results indicate that suitable intervention need to be tailored to regional differences in the southwest of the United States.

The Research Topic includes two articles that explore the impact of extreme heat on farm workers. El Khayat et al. provides a complete review of the state of the art of research related to heat, provide guidance as to the future research directions and inform policies aimed at protecting the health and safety of farmworkers, their analysis applies to migrant farmworkers as well and in the workplace setting. Their review results clearly identify the main topics currently under analysis by the academic community and others. Most of the literature reviewed focuses on health-related issues. Determinants of health outcomes include gender, environmental conditions, healthy practices such as hydration while working, wearing appropriate clothing and others. Similarly, López-Carr et al. provides a framework for the study of joint or cumulative negative impacts resulting from the joint and/or concurrent occurrence of heat and COVID-19 and due to policies aimed at reducing both COVID-19 and heat impacts in the workplace. López-Carr et al. find that policies that are aimed at preventing the occurrence of COVID-19 may not be compatible with those aimed at preventing the negative impacts caused by heat stress.

Results suggest that the opposite can also be true. The article suggests ways that this apparent conflict between on-the-job regulations can be reduced so that farm workers can benefit from a healthier job environment. Equally important is the fact that all farm workers are exposed to more than one environmental threat at a time such as heat and chemical exposure. Thus, López-Carr et al. open an important line of research inquiry that could result in improvements on the wellbeing of farm workers.

To conclude, the Research Topic presents a wide perspective on what socio-economic drivers have an impact on migrant farm workers' health. The Research Topic is rich on methods and empirical findings and present conceptual notes for further research. In addition, the Research Topic addresses issues related to both off-farm and on-farm determinants of health. What is clear from the articles featured in the Research Topic is that farm worker health is very specific to geographical context, and that policies aimed at improving farm worker social welfare need to consider geographical context coupled with other factors such as gender, ethnicity and other aspects of the cultural heritage of migrant and non-migrant farmworkers.

## Author contributions

FC: Conceptualization, Investigation, Writing – original draft, Writing – review & editing. DL-C: Conceptualization, Investigation, Writing – review & editing. AS: Conceptualization, Investigation, Writing – review & editing.
